# Metabolic acidosis due to ingestion of lanthanum sulfates and chlorides in cetyl alcohol solution pool cleaner

**DOI:** 10.1002/ccr3.4121

**Published:** 2021-05-05

**Authors:** Kaushik Ivaturi, Antoney J. Ferrey, Lawrence Nguyen, Sam Tonthat, Sanjivan Kohli, Everado Arias Torres, Kamyar Kalantarzadeh, Ramy Hanna

**Affiliations:** ^1^ Department of Medicine‐Division of Nephrology University of California Irvine Orange CA USA; ^2^ Department of Medicine‐Division of Critical Care and Pulmonary Medicine University of California Irvine Orange CA USA; ^3^ Department of Medicine University of California Irvine Orange CA USA

**Keywords:** alcohol dehydrogenase, anion gap metabolic acidosis, continuous renal replacement therapy, emergency ingestion, hyponatremia, osmolal gap, toxic alcohol, toxicology

## Abstract

Cetyl Alcohol is a rare cause of acidosis if ingested in large quantities. Hyponatremia with overlapping anion gap and osmolal gap‐positive metabolic acidosis may appear to have iso‐osmolar serum. This is a case of an unusual toxic exposure.

## Graphical abstract

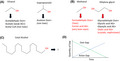

## 1 INTRODUCTION

A 67‐year‐old female ingested lanthanum chloride/sulfate in cetyl alcohol phosphate remover. Hyponatremia with a sodium of 119 meq/L and profound osmolal gap‐positive metabolic acidosis (pH = 6.99, HCO_3_ = 9) resulted. Measured serum osmolality was 290 mOsm/L due to a positive anion gap and osmolal gap acidosis overlapping with true hypoosmolar hyponatremia.

The diagnosis of anion gap metabolic acidosis is filled with emergent exposures that benefit from prompt medical diagnosis and immediate intervention.^1^ The toxicity of certain exposures depends on the end products of metabolism of the ingested alcohol by human alcohol dehydrogenase in hepatocytes.^2^, ^3^ Ethanol for instance is broken down to acetaldehyde and acetate, generating only a minor anion gap. In the case of isopropyl alcohol, it is ultimately metabolized to acetone.^4^ These biochemical changes substantiate why these alcohols merely generate an osmolal gap without acidosis and clinically only present with inebriation.^2^

Other alcohols, like ethylene glycol and methanol, are metabolized to oxalic acid and formic acid, respectfully.^5^ This ultimately leads to oxalate urinary crystallization, anion gap metabolic acidosis, and renal failure in the case of ethylene glycol.^5^ Similarly, methanol metabolism results in formic acid formation causing blindness, anion gap metabolic acidosis, and some degree of renal compromise.^6^ Toluene is metabolized to hippuric acid, an anion which is freely filtered and excreted in individuals with normal renal function, and as a result toluene toxicty presents as a nonanion gap metabolic acidosis. In individuals with chronic kidney disease, toluene may present with an anion gap metabolic acidosis.^7^

**FIGURE 1:**
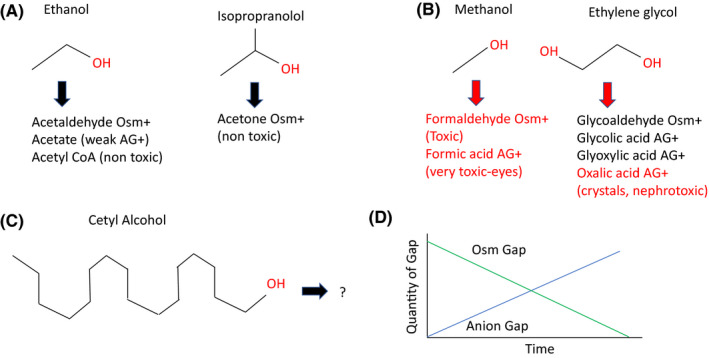
Metabolism of alcohols. +, Present; AG, anion gap; Osm, serum osmolal gap. A, Nontoxic alcohol metabolism. B, Toxic alcohol metabolism. C, Cetyl alcohol, the organic alcohol ingested. D, Relationship between anion gap and osmolal gap in a toxic alcohol ingestion, vs time

Close attention is required to patients with severe metabolic acidosis, and though an anion gap is helpful in suspecting and making the diagnosis, the appearance of an anion gap depends on the timing of the alcohol ingestion. Biochemically, toxic alcohols are first converted to a carboxylic acid, lowering the pH. Then, the carboxylic acid is converted to a ketone or aldehyde as it is eliminated and deactivated. Figure 1 demonstrates this for the metabolism of toxic alcohol, and Table 1 reviews the differential diagnosis of high anion gap metabolic acidosis. These data will be presented in more depth in our presentation of a patient who became ill after an unusual toxic alcohol exposure.

**TABLE 1:** Differential diagnosis of high anion gap anion acidosis

Cause
Methanol
Ethylene glycol, propylene glycol
5‐Oxo‐proline (Chronic acetaminophen use in high doses)
L‐lactate acidosis (Ethanol, ischemia, metformin, isoniazid, linezolid, and benzodiazepine infusion syndrome)
D‐lactate short gut syndrome
Methanol
Salicylates
Organic acids and uremic toxins in acute kidney injury
Diabetic Ketoacidosis
Starvation Ketoacidosis
Alcoholic ketoacidosis
Cyanide
Carbon Monoxide
Aminoglycosides
Toluene toxicity (hippuric acid, hippurate)‐NAGMA in normal renal function AGMA only in CKD patients

Abbreviations: AGMA, anion gap metabolic acidosis; CKD, chronic Kidney disease; NAGMA, nonanion gap metabolic acidosis.

## 2 CASE REPORT

We report a case of a 67‐year‐old relatively healthy female who was brought in by emergency medical services obtunded after family members reported that she ingested an unidentified brown liquid. The family suspected that the patient, who was depressed, had done so as a suicide attempt. Her typical medications did not include Metformin or high‐dose aspirin. She however reportedly did drink an ethanol‐based beverage before the ingestion in question. There was no suspected glue sniffing, ethylene glycol was not easily accessible to the patient, and no methanol was on the premises of the property per the patient's family. The type of liquid ingested was initially unknown. In emergency department, alcohol screen was positive at 188 meq/L.

The patient's pH was 6.99‐7.05, and her serum bicarbonate as measured on chemistry laboratories was 9‐11 meq/L. The partial pressure of carbon dioxide (PaCO_2_) on the blood gas was 28.6 mm Hg. The expected PaCO_2_ by Winter's formula (1.5 * [HCO_3_] + 8 ± 2) was 21.5 ± 2 mm Hg, this indicating a mild secondary respiratory acidosis. The anion gap noted (AG) on admission was 19‐21 points with an albumin of 4.1 g/dL on day 1, dropping to 2.5 g/dL the next day. Anion gaps were corrected for serum albumin when appropriate. Serum sodium was noted to be 119 meq/L, potassium was 3.3 meq/L, chloride was 89 meq/L, and serum creatinine was within normal range at 0.7 mg/dL. Lactic acid was not elevated at 1.7 meq/L.

Measured serum osmolality was remarkably normal between 287 and 290 mSOm/L. Pseudohyponatremia was considered, but her serum glucose was 99 mg/dL, triglycerides were 111 mg/dL, low‐density lipoprotein (LDL) was low at 17 mg/dL, and total cholesterol was 96 mg/dL. Total protein was found to be 6.7 g/dL on day 1 and dropped down to 4.2 g/dL on day 2. The total protein–albumin calculation for globulin was 1.7‐3.7 g/dL and was never elevated. There was no M‐Spike found, and she had not received any intravenous immunoglobulin (IVIG) that could induce pseudohyponatremia from a globulinemia. Of note, serum phosphate was not low, at 4.2 mg/dL initially but dropped to 1.7‐2.1 later (after being started on renal replacement therapy). Serum potassium also later dropped to 1.7‐2.6 meq/L after being started on renal replacement therapy as well.

Serum sodium was 119 meq/L, and the patient's serum osmolality as mentioned was normal, and workup for pseudohyponatremia was not suggestive of a cause of dilutional hyponatremia. Glucose, triglycerides, cholesterol, and serum total protein were all within normal range. Urine sodium was 79 meq/L and urine osmolality was 331 mOsm/L, and at this point, the data suggested a true hypoosmolar hyponatremia with secondary osmolal gap‐positive and anion gap‐positive metabolic acidosis from ingestion. The pattern of hyponatremia appeared to be due to a syndrome of inappropriate ADH secretion (SIADH)‐based mechanism by urine electrolytes.

The osmolal gap was calculated based on the concern for a circulating organic anion, rather than the chance of this patient presenting with a pseudohyponatremia which had been carefully excluded as previously noted. The calculated serum osmolality ranged from 248 to 255 mOsm/L, and the calculated serum osmolal gap was 37.5‐40 mOsm/L.

**FIGURE 2:**
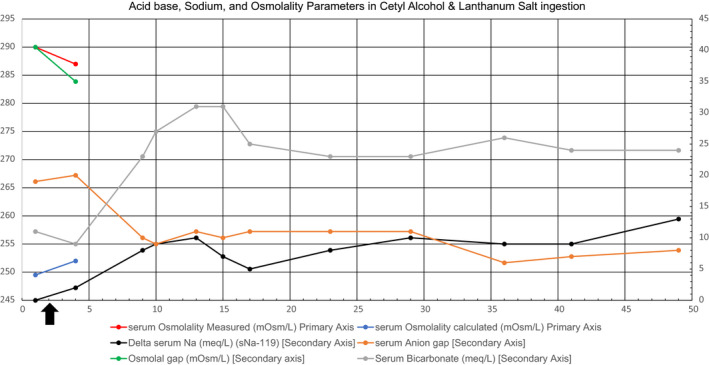
Acid base, sodium, and osmolality parameters in cetyl alcohol and Lanthanum salt ingestion. Black arrow, start of continuous renal replacement therapy (CRRT). Meq/L, milliequivalent/Liter; mOsm/L, milliosmoles/Liter; sNa, serum sodium

Figure 2 shows trends of pH, serum bicarbonate, serum sodium, serum anion gap, and serum osmolal gap as the metabolic derangements stemming from the ingestion were treated. The calculated serum osmolality was far lower.

The patient was given fomepizole immediately (15 mg/Kg) to inhibit alcohol dehydrogenase. In addition, high doses of thiamine, folic acid, and pyridoxine cofactors were administered. Folic acid was given 50 mg intravenously (IV) every 6 hours, thiamine was given 100 mg IV daily, and pyridoxine was given 50 mg IV daily as toxic alcohol was not identified. Cofactor administration was intended only as a temporizing therapy meant to push the metabolites toward alternative enzymatic pathways that do not produce toxic metabolites. As such, concurrent with above therapy, the patient was being prepared for hemodialysis (renal replacement therapy—RRT), with access placement.

Given the patient's hypotension with systolic blood pressure of 90 mm Hg, and need for ongoing vasopressor support, continuous renal replacement therapy (CRRT) was pursued over conventional single‐pass intermittent hemodialysis. The patient's serum sodium was corrected carefully using a modified mixture of pre‐ and postfilter fluids, dialysate, and replacement fluid to maintain appropriate tonicity. The initial prescription was comprised of 0.9% normal saline at 200 mL/hour prefilter, 500 mL/hour of 0.9% normal saline postfilter, and prismasate dialysate with a sodium concentration of 140 meq/L at 1000 mL/hour. The replacement fluid was initially run as sterile water +1.5 amps (75 meq/L) of sodium bicarbonate (NaHCO_3_) at 1000 mL/hour. The initial delivered sodium concentration of this CRRT was 119 meq/L of therapy fluid given. The bicarbonate drip was then slowly adjusted over the next 24 hours to keep serum sodium from precipitous increase. As sodium increased, the bicarbonate drip was increased to 2 amps 100 meq/L, increasing the delivered serum Na concentration of CRRT to 128.8 meq/L. As a result, the serum sodium corrected appropriately to 127 meq/L by 24 hours (a 24‐hour delta sodium of 8 meq/L) and by 48 hours it had corrected to a goal of 132 meq/L (a 48‐hour delta sodium of 13 meq/L).

The pH corrected to 7.4 by 30 hours and CRRT was discontinued at 48 hours when it became apparent that the patient's renal function remained intact, pH had corrected, anion gap had returned to normal (AG = 10), and the serum sodium normalized. The patient had ongoing neurological workup, treatment for aspiration pneumonitis, and lung injury from the surfactant qualities of the ingested fluid requiring prolonged intubation and tracheostomy placement. This ingestion ultimately led to prolonged intensive care and transfer to a long‐term acute care facility upon discharge. Please see Figure 2 for trends of serum sodium pH, serum bicarbonate, anion gap, measured serum osmolality, calculated serum osmolality, and osmolal gap.

This case poses a different sort of alcohol ingestion than that which is commonly encountered clinically. Cetyl alcohol is commonly used in cosmetic applications,^8^ and the toxic dose is postulated to be at >5 g/Kilogram, but cases of ingestion are very rare. In this case, the patient drank a large amount of fluid from a 3 L container of phosphorous remover. The cleaning product was comprised of Lanthanum chloride, Lanthanum sulfate, and cetyl alcohol. The concentration of cetyl alcohol was 4% (4 g/100 mL), and assuming full ingestion, she could have ingested up to 120 g from the 3 L bottle. The patient weighed 49.9 kg, and the 50% lethal dose of cetyl alcohol is 125 g (5 g/Kg). This indicates the amount of cetyl alcohol ingested was significant enough to be considered lethal in a human subject. The patient's neurological disturbances can be directly attributed to cetyl alcohol.^8^

## 3 DISCUSSION

The presence of an anion gap is an early clinical finding after alcohol exposure. Later, an osmolal gap and anion gap may overlap as the acidic metabolite is converted into an aldehyde/ketone. Finally, the acidic intermediary is eliminated leaving only an osmolal gap. The clinical management depends on the two pronged approach of redirection or inhibiting alcohol dehydrogenase, preventing formation of toxic metabolites.^9^ The other approach is to remove toxic alcohols from circulation which typically involves hemodialysis to remove the toxic exposure and correct the accompanying metabolic derangements.^9^

Generally, the clinical importance of diagnosing or suspecting an anion gap metabolic acidosis is to generate a differential that rapidly takes into account the deleterious effects of dangerous biochemical metabolites generated by metabolism of the ingested acid. In this case, we discuss an unusual exposure not usually associated with a metabolic acidosis, large‐chain fatty acid alcohols. These alcohols are atypically ingested in large amounts and not generally thought to cause an anion gap metabolic acidosis. We present this case for the specific causes of identifying future episodes of this exposure in the future and analyzing the biochemistry that could be behind such a surprising toxic ingestion.

It should be noted that the ingestion of Lanthanum salts, and sulfates, likely contributed to the acidosis and the overall toxicity observed. Ethanol may have contributed to the osmolal gap. In retrospect, it is difficult to distinguish the role of the cetyl alcohol from the sulfates and their respective contributions to the anion gap metabolic acidosis. It is known that cetyl alcohol is partially metabolized into palmitic acid, a nontoxic long‐chain fatty acid alcohol. What remains unknown is whether another acidic toxic metabolite developed in this particular patient that also contributed to the profound anion gap metabolic acidosis. It is possible that ketone bodies resulted from further metabolism of the long‐chain fatty acids generated by cetyl alcohol,^10^ but this requires further investigation.

## 4 CONCLUSION

This report discusses a unique, hitherto unreported ingestion that resulted in a complex pattern of metabolic derangements. This case reiterates the importance of recognizing that a gap acidosis in conjunction with hypoosmolar hyponatremia can result in a picture similar to pseudohyponatremia. This is a unique case that captures a severe ingestion episode that captured an unusual exposure to a rarely seen toxin. It is hoped that this report will serve as an archetype of this exposure, and allow similar cases to be readily suspected and recognized in the future.

### 4.1 Key learning points

1. Anion gap metabolic acidosis is crucial to diagnose early to prevent end organ damage morbidity and mortality.
2. A good history and corroboration with family members of a patient are helpful to identifying the toxic ingestion.
3. Cetyl alcohol containing pool cleaners if ingested in extreme quantities (liters) have the potential to form organic alcohols and negatively charged carboxylic acids that can result in hyponatremia, and anion gap metabolic acidosis.

## CONFLICT OF INTEREST

None.

## AUTHOR CONTRIBUTIONS

KI: wrote case report. AJF: edited text. LN: edited text. ST: edited text. SK: edited text. EAT: edited text. KK: edited text extensively. RH: oversaw writing of manuscript.

## ETHICAL APPROVAL

This research work does not contain human subject research material, as it is an individual anonymized case report. IRB permission was not applied for as it is not required for individual case reports.

## SPONSORSHIP

This work was not sponsored.
